# *Opuntia ficus-indica* Alleviates Particulate Matter 10 Plus Diesel Exhaust Particles (PM10D)—Induced Airway Inflammation by Suppressing the Expression of Inflammatory Cytokines and Chemokines

**DOI:** 10.3390/plants11040520

**Published:** 2022-02-14

**Authors:** Young-Sil Lee, Won-Kyung Yang, Ye-Rin Park, Yang-Chun Park, In-Jae Park, Geung-Joo Lee, Hyung-Sik Kang, Bong-Kyun Kim, Seung-Hyung Kim

**Affiliations:** 1 AtoGen Co., Ltd., Daejeon 34015, Korea; rheeys04@gmail.com; 2 Institute of Traditional Medicine and Bioscience, Daejeon University, Daejeon 34520, Korea; ywks1220@dju.kr (W.-K.Y.); dutndi@naver.com (Y.-R.P.); 3 Division of Respiratory Medicine, Department of Internal Medicine, College of Korean Medicine, Daejeon University, Daejeon 34520, Korea; omdpyc@dju.kr; 4 Hurum Co., Ltd. Room 416, Daeryung Techno-Town III, 115, Gasan Digital 2-ro, Geumcheon-gu, Seoul 08505, Korea; fungi1978@hurumcorp.com; 5 Department of Horticulture, Chungnam National University, 99 Daehak-ro, Yuseong-gu, Daejeon 34134, Korea; gjlee@cnu.ac.kr; 6 Department of Smart Agriculture Systems, Chungnam National University, 99 Daehak-ro, Yuseong-gu, Daejeon 34134, Korea; 7 School of Biological Sciences and Technology, Chonnam National University, 77 Yongbong-ro, Buk-gu, Gwangju 61186, Korea; kanghs@chonnam.ac.kr

**Keywords:** airway inflammation, *Opuntia ficus-indica*, particulate matter, diesel exhaust particle, PM10D-induced respiratory disease

## Abstract

Particulate matter (PM) exposure may cause adverse health effects such as respiratory disorders. We evaluated the protective effects of various *Opuntia ficus-indica* (OFI) extracts on airway inflammation associated with exposure to PM10D with an aerodynamic diameter <10 μm (PM10) and diesel exhaust particles (DEP). BALB/c mice were exposed to PM10D via intranasal tracheal injection three times over a period of 12 days and various OFI extracts (water, 30% ethanolic, or 50% ethanolic extracts) were administered orally for 12 days. All OFI extracts suppressed neutrophil infiltration and the number of immune cells (CD3^+^/CD4^+^, CD3^+^/CD8^+^, and Gr-1^+^/CD11b) in bronchoalveolar lavage fluid (BALF) and lungs. OFI extracts decreased the expression of cytokines and chemokines, including chemokine (C-X-C motif) ligand (CXCL)-1, interleukin (IL)-17, macrophage inflammatory protein-2, tumor necrosis factor (TNF)-α, cyclooxygenase-2, IL-1α, IL-1β, IL-5, IL-6, transient receptor potential cation channel subfamily V member 1, and mucin 5AC, and inhibited IRAK-1, TNF-α, and CXCL-1 localization in BALF and lungs of mice with PM10D-induced airway inflammation. Serum asymmetric and symmetric dimethyl arginine levels were also decreased by OFI extracts treatment. Moreover, all OFI extracts restored histopathological damage in the trachea and lungs of mice with PM10D-induced airway inflammation. These results indicate that OFI extracts may be used to prevent and treat airway inflammation and respiratory diseases.

## 1. Introduction

In 2016, the World Health Organization reported that 92% of people across the world live in areas where air pollution levels exceed safety limits. Exposure to air pollution causes various adverse health effects and increases the risks of mortality and morbidity associated with cardiovascular and respiratory diseases [1,2].

Particulate matter (PM), the principal component of air pollution, is a complex mixture of materials with a carbonaceous core, organic compounds, acids, and fine metal particles. PM is classified into coarse particles (aerodynamic diameter ≥ 2.5 μm and <10 μm), fine particles (diameter ≥ 0.1 μm and <2.5 μm,), and ultrafine particles (nanoparticles, diameter < 0.1 μm) [3]. The chemical composition of PM varies depending on its source [4] and PM with different physical properties may affect the respiratory system in different ways [5,6]. PM is deposited on the respiratory tract, lungs, and pulmonary alveoli, leading to inflammatory responses through stimulation of the immune system. This can lead to PM-mediated adverse health effects, such as respiratory system inflammation [7]. Exposure to ambient PM air pollution has adverse effects on the exacerbation, progression, and development of respiratory diseases such as asthma and chronic obstructive pulmonary disease (COPD) [8]. Despite increasing global interest in PM and related research, effective protective and therapeutic agents against PM remain to be identified. Therefore, there is a need for novel preventive and therapeutic agents for use against PM-induced respiratory damage.

*Opuntia ficus-indica* (L.) Mill. (OFI) belongs to the dicotyledonous angiosperm Cactaceae family and is commonly referred to as prickly pear or nopal cactus. It is native to North America but is widely distributed in the southern parts of the Korean peninsula, including diuretic, anticarcinogenic, anti-inflammatory, anti-diabetic, and anti-hypercholesterolemic properties [9]. It is therefore employed in the health, nutrition, and cosmetic industries, and has been used as an herbal remedy for diverse health problems in different countries [9]. According to many reports, OFI is known for its high content in polyphenols. The antioxidant and anti-inflammatory properties of polyphenols have been widely reported [9,10]. However, there are no reports of the inhibitory effects of OFI on airway inflammation induced by PM with an aerodynamic diameter < 10 µm (PM10), diesel exhaust particulates (DEP), and their combination. In the present study, we investigated whether administration of *OFI* could alleviate inflammation to protect the respiratory system in a PM10D-induced airway inflammation model in mice.

## 2. Results

### 2.1. Chromatographic Analysis of OFI Extracted with 50% Ethanol

**Figure 1:**
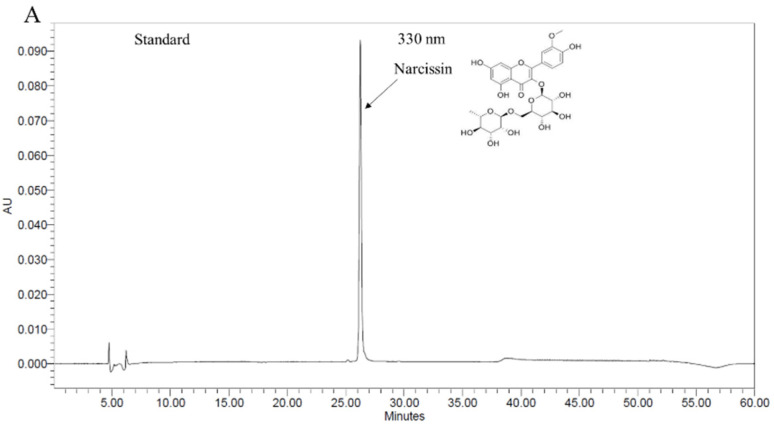

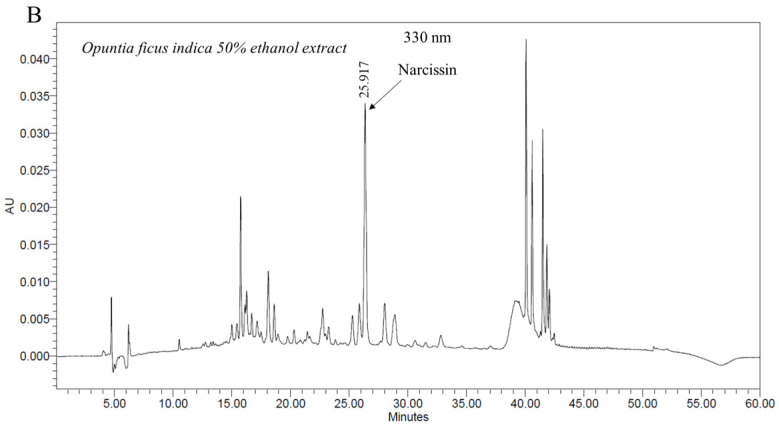
High-performance liquid chromatography (HPLC) chromatograms of a 50% ethanol extract of Opuntia ficus-indica (OFI) showing the structure of major compounds in the extract (**A**) Narcissin standard and (**B**) 50% ethanol extract of Opuntia ficus-indica.

Chromatograms of the 50% ethanol extract of OFI are shown in Figure 1. Narcissin was identified via HPLC, at a concentration of 1.59 ± 0.1 mg/g.

### 2.2. Effects of OFI-W, OFI-30E, and OFI-50E Administration on Immune Cell Numbers in BALF and Lung Tissues

**Figure 2:**
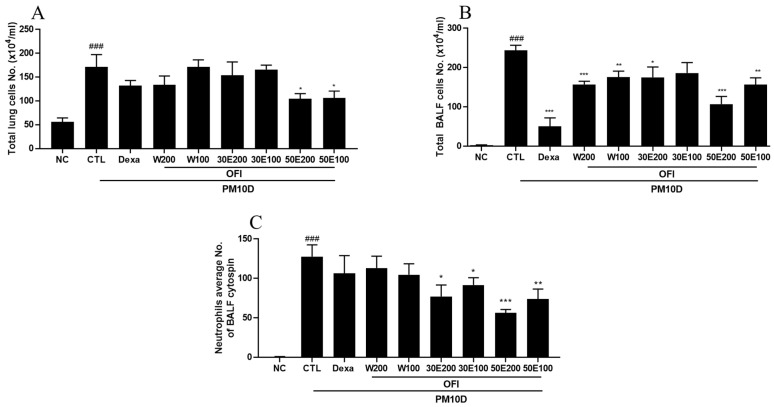

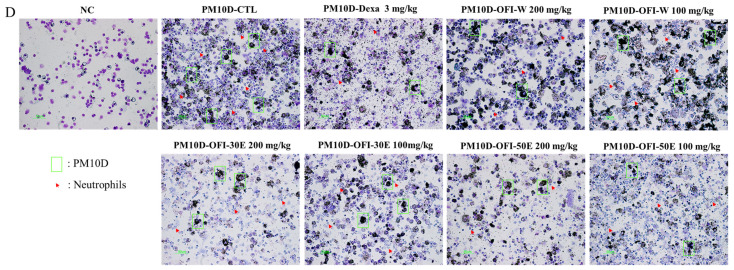
Effects of water and ethanolic extracts of OFI on airway immune cell numbers in in a PM10D-induced airway inflammation model. (**A**) Total lung cell numbers, (**B**) total bronchoalveolar lavage fluid (BALF) cell numbers, (**C**) neutrophils numbers in BALF cytospin, and (**D**) photomicrograph of BALF cytospins (magnification: 200×). NC: BALB/c normal control group; CTL: PM10D-induced control group; Dexa: 3 mg/kg dexamethasone-treated PM10D-induced group; OFI-W 100 and 200: PM10D-induced group treated with 100 and 200 mg/kg OFI-water extract, respectively; OFI-30E 100 and 200: PM10D-induced group treated with 100 and 200 mg/kg OFI-30% ethanol extract, respectively; OFI-50E 100 and 200: PM10D-induced group treated with 100 and 200 mg/kg OFI-50% ethanol extract, respectively. Data are expressed as means ± standard error of the mean (SEM), *n* = 8. ^###^ *p* < 0.005 vs. NC; * *p* < 0.05, ** *p* < 0.01, and *** *p* < 0.005 vs. CTL.

The total number of cells in the lungs was significantly decreased by administration of OFI-50E extract (Figure 2A). In addition, the total number of cells in BALF was decreased by administration of all OFI extracts and dexamethasone (Figure 2B). Mice treated with OFI-30E extract, OFI-50E extract, and dexamethasone showed significantly lower neutrophil infiltration than those in the PM10D-control group (Figure 2C,D). Moreover, the absolute number of CD3^+^/CD4^+^ and CD3^+^/CD8^+^ cells in the lungs was decreased by treatment with the OFI-50E extract, and the absolute number of neutrophils and Gr-1^+^/CD11b^+^ cells were decreased by administration of all OFI extracts, as shown via FACS analysis. Treatment with OFI also decreased the absolute numbers of these immune cells in BALF. These results indicate that the over-activation of the immune response caused by PM10D was suppressed by administration of OFI extracts (Table 1 and Table S1).

### 2.3. Effects of OFI-W, OFI-30E, and OFI-50E Administration on Inflammatory Cytokines in BALF

**Figure 3:**
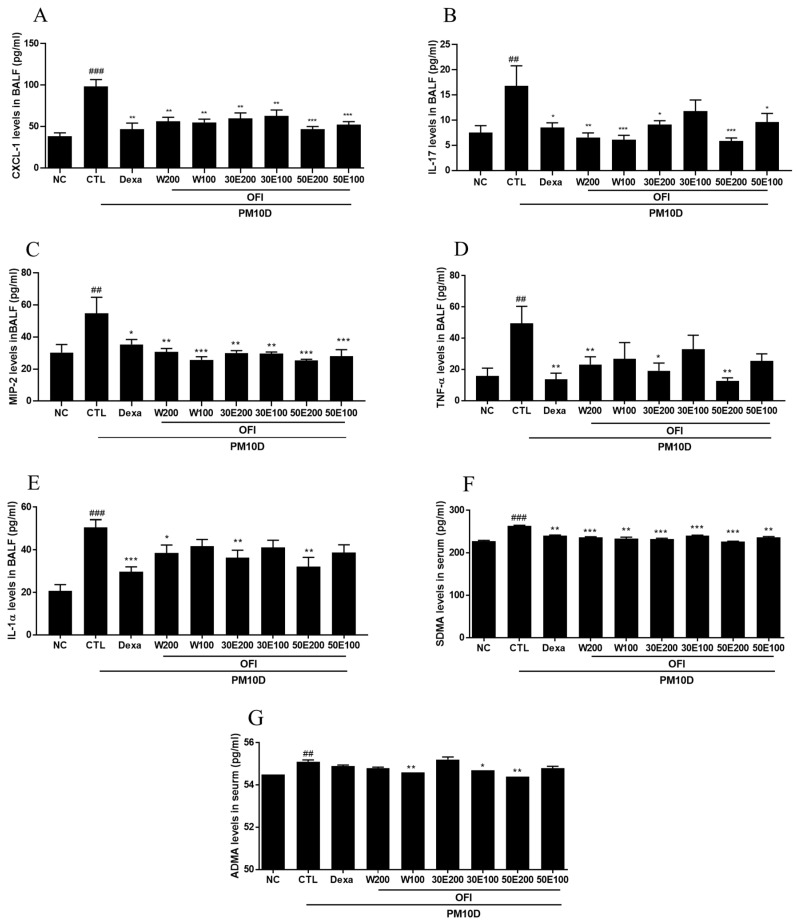
Effects of water and ethanolic extracts of OFI on pro-inflammatory cytokines in BALF and serum in a PM10D-induced airway inflammation model. (**A**) Chemokine (C-X-C motif) ligand 1 (CXCL-1), (**B**) interleukin (IL)-17, (**C**) macrophage inflammatory protein (MIP)-2, (**D**) tumor necrosis factor (TNF)-α, (**E**) IL-1α in BALF, (**F**) symmetric dimethyl arginine (SDMA), and (**G**) asymmetric dimethyl arginine (ADMA) concentrations in serum. NC: BALB/c normal control group; CTL: PM10D-induced control group; Dexa: 3 mg/kg dexamethasone-treated PM10D-induced group; OFI-W 100 and 200: PM10D-induced group treated with 100 and 200 mg/kg OFI-water extract, respectively; OFI-30E 100 and 200: PM10D-induced group treated with 100 and 200 mg/kg OFI-30% ethanol extract, respectively; OFI-50E 100 and 200: PM10D-induced group treated with 100 and 200 mg/kg OFI-50% ethanol extract, respectively. Data are expressed as means ± SEM, *n* = 8. ^##^ *p* < 0.01 and ^###^ *p* < 0.005 vs. NC; * *p* < 0.05, ** *p* < 0.01, and *** *p* < 0.005 vs. CTL.

Pro-inflammatory cytokines are secreted in response to inflammation and contribute to the pathology of respiratory diseases. As shown in Figure 3, CXCL-1, IL-17, MIP-2, TNF-α, and IL-1α levels in BALF in the PM10D-control group were higher than those of the NC group. CXCL-1 and MIP-2 levels were significantly suppressed by dexamethasone and administration of all OFI extracts compared with the PM10D-control group (Figure 3A,B). IL-17 levels were decreased in the dexamethasone and administration of all OFI extracts except OFI-30E100 extract-treated group compared with the PM10D-control group (Figure 3C). TNF-α and IL-1α levels were suppressed in the dexamethasone and OFI-W200, 30E200, and 50E200 groups compared with the PM10D-control group (Figure 3D,E).

### 2.4. Effects of OFI-W, OFI-30E, and OFI-50E Administration on ADMA and SDMA in Serum

ADMA and SDMA are associated with inflammation, endothelial dysfunction, and oxidative stress. As shown in Figure 3F,G, serum ADMA and SDMA concentrations were increased in the PM10D-control group compared with the NC group. Serum SDMA concentrations were decreased in all OFI extract-treated groups and dexamethasone, and serum ADMA concentrations were lowered in the OFI-W100, 30E200, and 50E200 groups compared with the PM10D-control group.

### 2.5. Effects of OFI-W, OFI-30E, and OFI-50E Administration on the Expression of Inflammatory Cytokines in the Lungs

**Figure 4:**
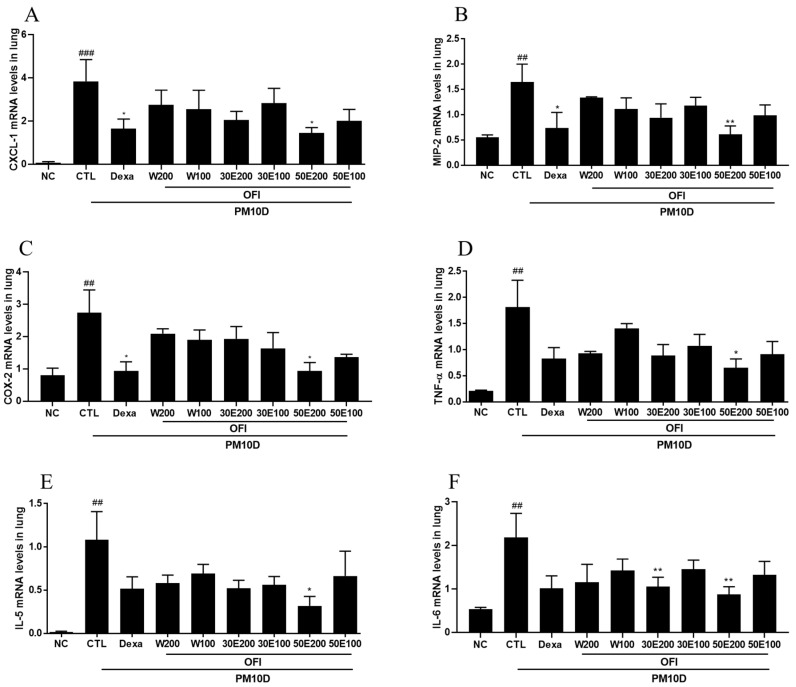

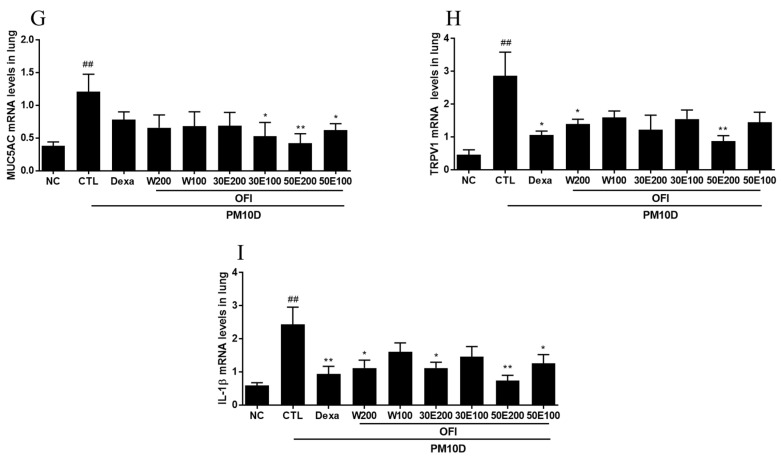
Effects of water and ethanolic extracts of OFI on cytokine gene expression in the lungs of mice with PM10D-induced airway inflammation. (**A**) CXCL-1, (**B**) MIP2, (**C**) cyclooxygenase (COX)-2, (**D**) TNF-α, (**E**) IL-5, (**F**) IL-6, (**G**) mucin 5AC (MUC5AC), (**H**) transient receptor potential cation channel subfamily V member 1 (TRPV1), and (**I**) IL-1β gene expression in the lungs. NC: BALB/c normal control group; CTL: PM10D-induced control group; Dexa: 3 mg/kg dexamethasone-treated PM10D-induced group; OFI-W 100 and 200: PM10D-induced group treated with 100 and 200 mg/kg OFI-water extract, respectively; OFI-30E 100 and 200: PM10D-induced group treated with 100 and 200 mg/kg OFI-30% ethanol extract, respectively; OFI-50E 100 and 200: PM10D-induced group treated with 100 and 200 mg/kg OFI-50% ethanol extract, respectively. Data are expressed as means ± SEM, *n* = 8. ^##^ *p* < 0.01 and ^###^ *p* < 0.005 vs. NC; * *p* < 0.05 and ** *p* < 0.01 vs. CTL.

CXCL-1, MIP-2, COX-2, TNF-α, and IL-5 mRNA expression in the lung was lowered by administration of the OFI-50E extract (Figure 4A–E). IL-6 and MUC5AC mRNA expression was reduced by treatment with OFI-30E and 50E extracts (Figure 4F,G). TRPV1 mRNA expression levels decreased after administration of OFI-W and 50E extracts, and IL-1β mRNA expression decreased in response to treatment with all OFI extracts (Figure 4H,I). In addition, dexamethasone treatment decreased the expression of these inflammatory cytokines in the lung. These results demonstrate that OFI extracts downregulated cytokine expression in the PM10D-control group.

### 2.6. Effect of OFI-W, OFI-30E, and OFI-50E Administration on IRAK-1, TNF-α, and CXCL-1 Expression

**Figure 5:**
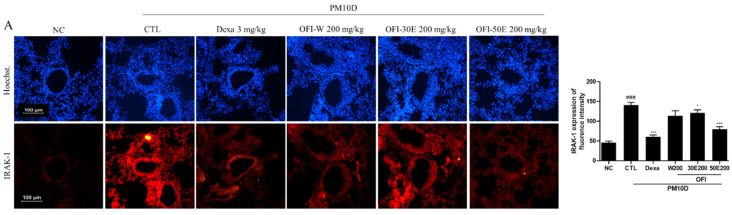

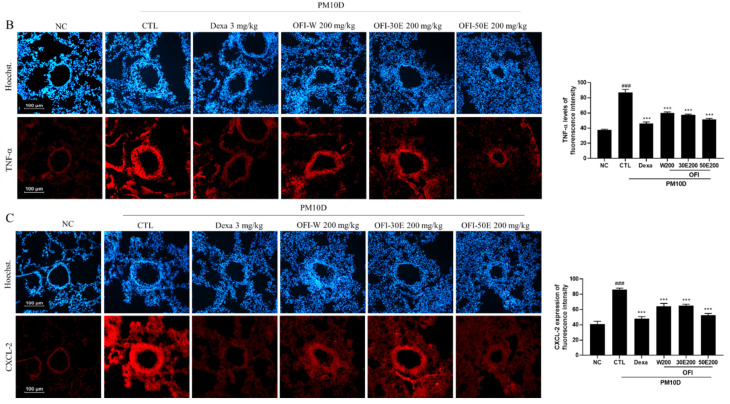
Immunofluorescence staining for interleukin-1 receptor-associated kinase 1 (IRAK1), TNF-α, and CXCL-1 protein expression in the lungs of mice with PM10D-induced airway inflammation. (**A**) IRAK1, (**B**) TNF-α, and (**C**) CXCL-1 expression in the lungs. NC: BALB/c normal control group; CTL: PM10D-induced control group; Dexa: 3 mg/kg dexamethasone-treated PM10D-induced group; OFI-W 100 and 200: PM10D-induced group treated with 100 and 200 mg/kg OFI-water extract, respectively; OFI-30E 100 and 200: PM10D-induced group treated with 100 and 200 mg/kg OFI-30% ethanol extract, respectively; OFI-50E 100 and 200: PM10D-induced group treated with 100 and 200 mg/kg OFI-50% ethanol extract, respectively. Data are expressed as means ± SEM, *n* = 8. ^###^ *p* < 0.005 vs. NC; * *p* < 0.05, *** *p* < 0.005 vs. CTL.

IRAK-1, TNF-α, and CXCL-1 play important roles in regulating inflammatory pathways. IRAK-1 expression was decreased in the lungs of mice treated with OPI-30E extract, OPI-50E extract, and dexamethasone. TNF-α and CXCL-1 expression was potently inhibited by treatment with all OPI extracts and dexamethasone. These results suggest that OPI extract treatment exerted anti-inflammatory effects on PM10D-induced airway inflammation (Figure 5A–C).

### 2.7. Effect of OFI-W, OFI-30E, and OFI-50E Administration on Lung Tissue Damage

**Figure 6:**
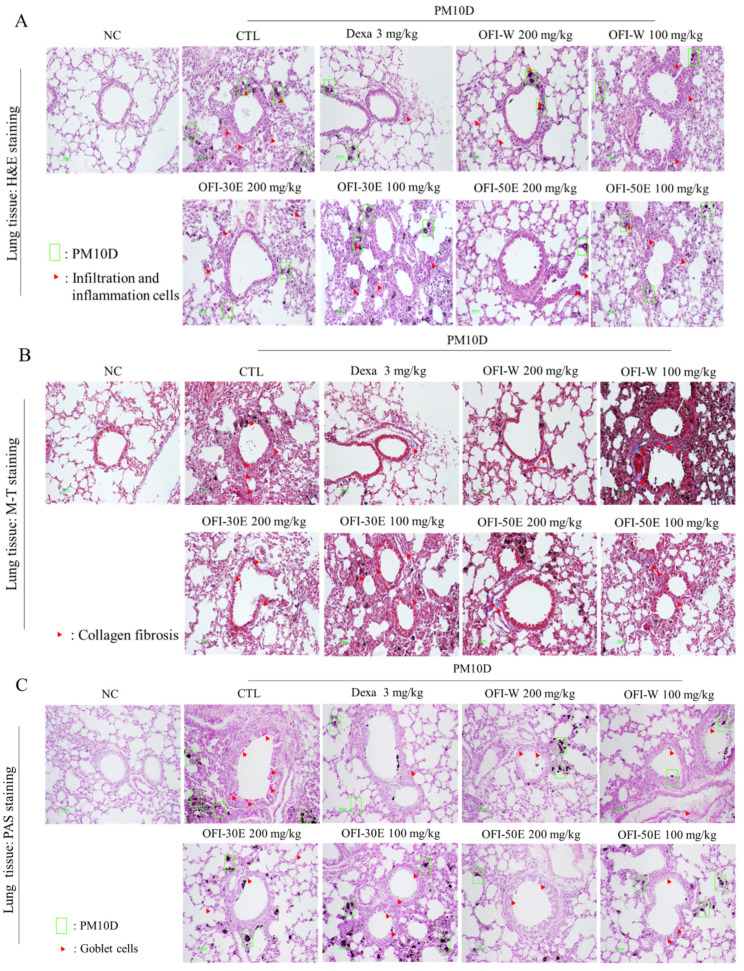

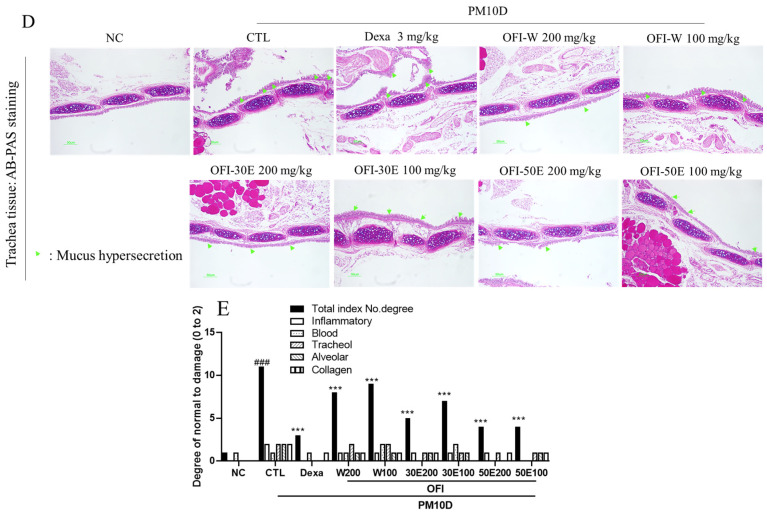
Effects of water and ethanolic extracts of OFI on lung and trachea histopathology in a PM10D-induced airway inflammation model. (**A**) Hematoxylin and eosin (H&E), (**B**) Masson’s trichrome (MT), (**C**) periodic acid-Schiff (PAS) staining of lung tissues, (**D**) Alcian blue (**A**,**B**)-PAS staining of tracheal tissue, and (**E**) quantitative analysis of the degree of lung tissue damage. BALB/c normal control group; CTL: PM10D-induced control group; Dexa: 3 mg/kg dexamethasone-treated PM10D-induced group; OFI-W 100 and 200: PM10D-induced group treated with 100 and 200 mg/kg OFI-water extract, respectively; OFI-30E 100 and 200: PM10D-induced group treated with 100 and 200 mg/kg OFI-30% ethanol extract, respectively; OFI-50E 100 and 200: PM10D-induced group treated with 100 and 200 mg/kg OFI-50% ethanol extract, respectively. Data are expressed as means ± SEM, *n* = 8. ^###^ *p* < 0.005 vs. NC; *** *p* < 0.005 vs. CTL.

During airway inflammation, infiltration of various immune cells, including neutrophils, lead to the destruction of alveolar cells, collagen deposition, and mucus secretion by goblet cells in the airways. Histological analysis of lung and tracheal tissues revealed that OFI extract, and dexamethasone treatment decreased histopathological scores, including the degree of inflammation around the bronchus and the mucus content in respiratory epithelial cells. These results indicate that OFI extracts prevented histopathological changes in the lung (Figure 6A–E).

### 2.8. Effects of PM10D on the Inflammatory Response in the Lung

**Figure 7:**
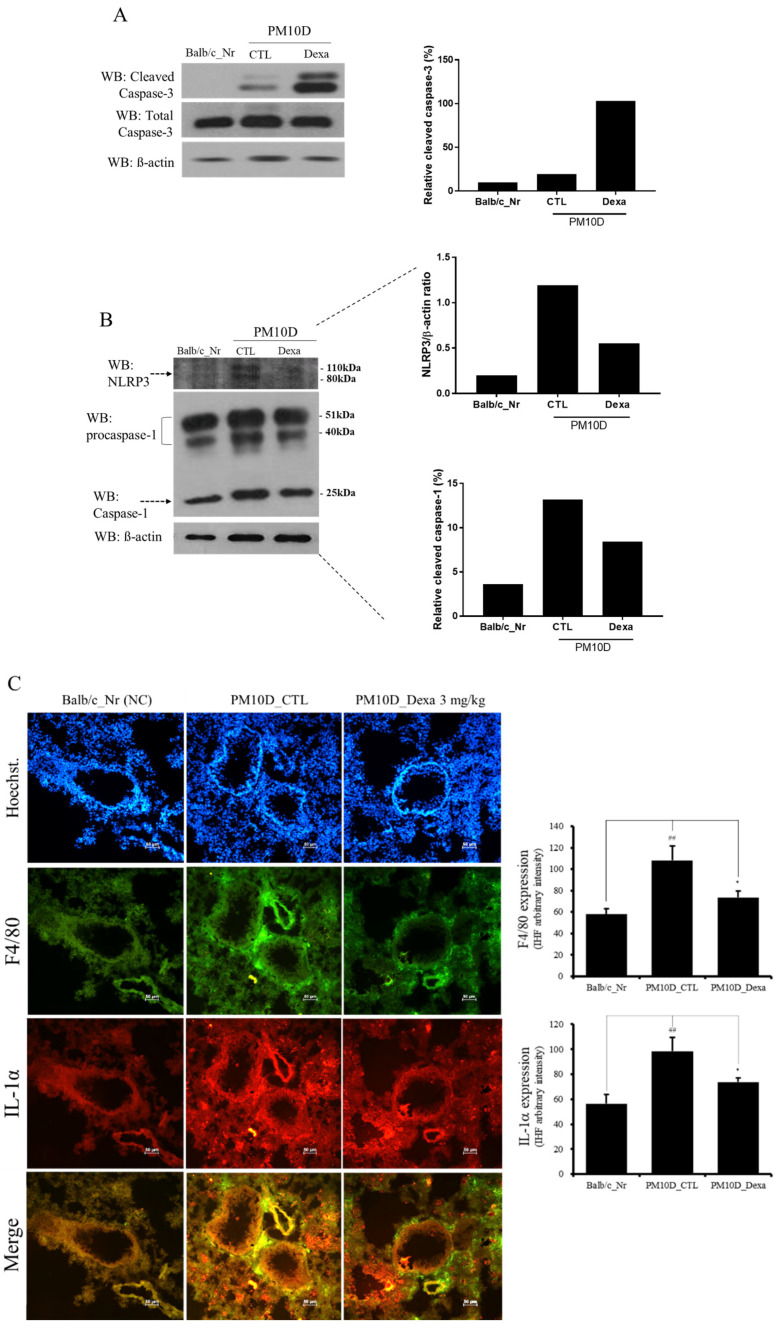
Effects of PM10D on inflammatory responses in the lung. (**A**) Caspase-1 expression, (**B**) NLR family pyrin domain containing 3 (NLRP3) and caspase-3 expression, and (**C**) immunofluorescence staining for F4/80 and IL-1α in the lung. BALB/c normal control group; CTL: PM10D-induced control group; Dexa: 3 mg/kg dexamethasone-treated PM10D-induced group. Data are expressed as means ± SEM, *n* = 5. ^##^ *p* < 0.01 vs. NC; * *p* < 0.05 vs. CTL.

Particle pollutants trigger and exacerbate inflammation. Inhaled fine particulates are engulfed by alveolar macrophages and induce alveolar macrophage cell death via pyroptosis. Dead cell-derived factors such as IL-1α/β are released and form inducible bronchus-associated lymphoid tissues (iBALT), which may contribute to particulate-pollution-induced inflammation [11,12]. During pyroptosis, caspase-1 expression and the formation of the NLRP3 inflammasome are increased [11]. To investigate the effects of dexamethasone on PM10D-induced airway inflammation, inflammatory cell death (pyroptosis) was evaluated in PM10D-exposed mice receiving dexamethasone. Expression of cleaved caspase-3 was increased in the lungs of PM10D-exposed mice receiving dexamethasone compared to that in untreated PM10D-exposed mice (Figure 7A). PM10D also increased the expression of NLRP3 and caspase-1 in the lung, which was suppressed by the administration of dexamethasone (Figure 7B). Moreover, PM10D increased the expression of the macrophage marker F4/80 and IL-1α in the lungs (Figure 7C). These results indicate that PM10D induces inflammatory alveolar macrophage death via pyroptosis and not apoptosis.

## 3. Discussion

All living organisms are increasingly being exposed to fine dust particles associated with industrial and urban development. This increases the risk of PM-induced chronic diseases and there have been attempts to develop preventive and therapeutic strategies against such diseases. In this study, we examined whether administration of OFI extract could exert respiratory protective effects in a PM10D-induced airway inflammation model.

Exposure to PM and DEP triggers inflammation with a unique immune response in the airways and lungs, as these particulates can move through the airways and deposit in the alveolar region. There, alveolar macrophages perform phagocytic activities by releasing cytokines and chemokines [13,14]. Alveolar macrophage death occurring via the programmed cell death process of pyroptosis, IL-1α/β release, and subsequent iBALT formation play important roles in the immune response occurring specifically in the lung. In addition, pyroptotic cells increase caspase-1 and NLRP3 inflammasome expression [11,12,15]. The NLRP3 inflammasome is a major regulator of inflammation through its activation of pro-caspase-1, which cleaves pro-1β into its mature form IL-1β, a critical proinflammatory cytokine that controls the severity of inflammation. In the present study, we showed that PM10D induced the expression of caspase-1 and that of the NLRP3 inflammasome with IL-1 α, indicating that PM10D-induced inflammation is related to pyroptosis.

PM activates innate immune cells but may also modify the function of T cells or cause direct cell-cell or cytokine interactions with T cells [7], which are key players in pulmonary inflammation. Furthermore, IL-1 α/β is recognized by the IL-1 receptor (IL-1R) as a ligand and activates IRAKs. IRAKs are key activators of the nuclear factor (NF)-κB and mitogen-activated protein kinase (MAPK) pathways, leading to the regulation of inflammatory cytokines [16]. This has been demonstrated in many studies [17,18,19]. Consistent with previous reports, in the present study, we found that various OFI extracts decreased the numbers of neutrophils and various immune cells, including CD3^+^/CD4^+^, CD3^+^/CD8^+^, and Gr-1^+^/CD11b^+^ in the lungs and BALF. In addition, various OFI extracts showed decreased concentrations of CXCL-1, IL-17A, MIP2, and TNF-α in BALF, but also suppressed the expression of many pro-inflammatory cytokines, including CXCL-1, MIP2, COX-2, TNF-α, IL-5, IL-6, IL-1β, and TRPV1 in the lungs of mice with PM10D-induced airway inflammation. Furthermore, various OFI extracts showed reduced localization of IRAK-1, TNF-α, and CXCL-1 in the lungs of mice with PM10D-induced airway inflammation. These results indicate that various OFI extracts effectively suppressed PM10D-induced immune activation, thus decreasing inflammation. Treatment with OFI-50E was more effective than that with other extracts.

Airway inflammation induced by PM and DEP exposure causes various histopathological changes in the lungs and trachea, with increased infiltration of inflammatory cells, fibrosis, mucus production, and epithelial thickness [20]. ADMA is an endogenous inhibitors of nitric oxide synthesis and SDMA is a competitor of transport for L-arginine to produce nitric oxide (NO). They are able to inhibit the synthesis and/or availability of NO that is required for normal lung function. In the lung, NO plays a role in airway and vascular smooth muscle relaxation, ventilation perfusion matching, mucociliary clearance and airway mucus secretion. Also, higher ADMA level also results in enhanced arginase activity, thereby contributing to collagen synthesis in the airway to the evolution of lung fibrosis. ADMA and SDMA levels in serum are increased in COPD and further elevated during an acute exacerbation [21,22,23]. MUC5AC increases mucus production, and increased mucus production followed by stimulated secretion, which may cause histological changes such as mucus plugging and airway obstruction [23,24,25]. In the present study, we showed that OFI extracts reduced airway inflammation, collagen fibrosis, goblet cell hyperplasia, and mucus hypersecretion in the lungs and trachea. Also, serum ADMA and SDMA concentrations and MUC5AC gene expression were reduced by OFI extracts. These results suggest that OFI extracts may contribute to regulation of an airway remodeling with reduced airway inflammation and may protect against PM10D-induced histopathological changes in the lungs and trachea.

## 4. Materials and Methods

### 4.1. Preparation of OFI Extract

Succulent and spatulate stems of OFI were provided by Biodiversity Research Institute, Jeju Technopark (voucher specimens No. 20080711006). and They were collected on Jeju Island, Republic of Korea (Latitude 33.24588, Longitude 126.23090), and were authenticated by Professor Geung-Joo Lee at Chungnam National University, Daejeon, Korea. The harvested stems were washed and freeze-dried for 48h in a freeze dryer (FD5518, Ilshin Co., Yangju, Korea). The dried OFI stems were ground using a grinder (SMX-4000DY, Shinil Co., Cheonan, Korea) for 1 min to pass through a100-mesh screen. The ground OFI stems were subjected to extraction with eight volumes of water, 30% ethanol, or 50% ethanol for 5 h at 60 °C. The extracts were then concentrated under reduced pressure using a rotary evaporator (Buchi R-114 Rotary evaporator with Buchi B-480 heating Bath, Flawil, Switzerland), suspended in distilled water to >20 Brix, and dried to obtain a powder by a freeze drier (Freeze dryer, FDU-540, Eyela, Tokyo, Japan).

### 4.2. High-Performance Liquid Chromatography (HPLC) Analysis of a 50% Ethanolic Extract of OFI (OFI-50E)

OFI-50E (250 mg) was dissolved in 80% methanol and filtered through a polyvinylidene difluoride syringe filter. HPLC analysis was performed using a Waters Alliance 2695 with 996 photodiode array detector system (Waters Corp., Milford, MA, USA). Samples (10 µL) were separated on a ShiseidoCapcell Pak C18 column MGII (5 μm, 4.6 × 250 mm, 40 °C) at a flow rate of 0.6 mL/min and eluted using a linear gradient of two mobile phases (A, ultrapure water; B, acetonitrile containing 0.1% trifluoroacetic acid). A chromatographic gradient was optimized as follows: 0 min, 90% A; 0–10 min, 70% A; 10–15 min, 67% A; 15–32 min, 60% A; 32–36 min, 0% A; 36–45 min, 0% A; 45–50 min, 90% A; 50–60 min, 90% A. All extraction and chromatographic solvents used were of mass spectrometry grade (Billerica, MA, USA). Narcissin was purchased from Sigma-Aldrich (St. Louis, MO, USA).

### 4.3. Animals and Treatments

Male BALB/c mice (6–8 weeks old) were purchased from Orient Bio Co., Ltd. (Seongnam-Si, Gyeonggi-do, Korea). The mice were housed at 21 ± 2 °C, with a relative humidity of 60 ± 10%, and under a 12-h light/dark cycle. Food and water were provided ad libitum. The study protocol was approved by the Committee for Animal Welfare at Daejeon University (DJUARB2019-021) and was performed in accordance with the committee guidelines. Mice were orally administered various OFI extracts every other day for 12 days, and received intranasal administration of a fine dust complex solution (PM10, ERM CZ-120; DEP, SRM 2975, Sigma-Aldrich, St. Louis, MO, USA) on days 4, 7, and 10. PM10 (3 mg/mL) and DEP (0.6 mg/mL) were dissolved in 1% aluminum hydroxide gel adjuvant and 99% saline. The mice were divided into nine treatment groups (*n* = 8 per group): (1) non-treated control (BALB/c normal, NC); (2) PM10+DEP-sensitized control (PM10D-CTL); (3) positive control, 3 mg/kg dexamethasone-treated PM10 + DEP-sensitized mice (PM10D-Dexa); (4) 200 mg/kg OFI water extract-treated PM10 + DEP-sensitized group (PM10D-OFI-W 200 mg/kg); (5) 100 mg/kg OFI water extract-treated PM10 + DEP-sensitized group (PM10D-OFI-W 100 mg/kg); (6) 200 mg/kg OFI 30% ethanol extract-treated PM10 + DEP-sensitized group (PM10D-OFI 30E 200 mg/kg); (7) 100 mg/kg OFI 30% ethanol extract-treated PM10 + DEP-sensitized group (PM10D-OFI 30E 100 mg/kg); (8) 200 mg/kg OFI 50% ethanol extract-treated PM10 + DEP-sensitized group (PM10D-OFI 50E 200 mg/kg); and (9) 100 mg/kg OFI 50% ethanol extract-treated PM10 + DEP-sensitized group (PM10D-OFI 50E 100 mg/kg). Mice in the positive control group were administered 3 mg/kg dexamethasone (Supplementary Figure S1). The general condition of mice, including physical appearance, behavior, hair condition, liveliness, sensitivity, and respiratory conditions, were checked daily. On day 12, all mice were euthanized, and blood, bronchoalveolar lavage fluid (BALF), and lung and trachea tissues were collected for further experiments.

### 4.4. Collection of Lung Cells and BALF and Cytological Analysis

Lungs were rinsed, minced, and incubated in phosphate-buffered saline (PBS) containing 1 mg/mL collagenase IV (Sigma-Aldrich, St. Louis, MO, USA) at 37 °C for 40 min. The cell suspension was then filtered and centrifuged at 450× *g* for 20 min, and cell pellets were collected. BALF from the trachea and lungs was obtained via tracheal cannulation after tracheotomy and centrifuged (400× *g*, 5 min, 4 °C). Collected lung cells and BALF were suspended in PBS, and total cell numbers were determined using a hemocytometer and then used to analyze the fluorescence-activated cell sorting (FACS). For cytological analysis, cells from BALF were centrifuged (400× *g*, 4 min) onto cytospin slides, fixed, stained with a modified Diff-Quik stain, and differential cell counts were determined.

### 4.5. Measurement of Inflammatory Mediators in BALF and Serum

Expression of chemokine (C-X-C motif) ligand 1 (CXCL-1), macrophage inflammatory protein 2 (MIP2), tumor necrosis factor-α (TNF-α), interleukin (IL)-17A, and IL-1 α in BALF, and serum concentrations of asymmetric dimethylarginine (ADMA) and symmetric dimethyl-arginine (SDMA) were determined using enzyme-linked immunosorbent assay (ELISA) kits according to the manufacturer’s instructions (R&D Systems, Minneapolis, MN, USA).

### 4.6. Flow Cytometry

Cells from the lungs and BALF were incubated with anti-CD3, anti-CD4, anti-CD8, anti-CD11b, and anti-granulocytic marker Gr-1 antibodies for 30 min, washed with PBS, and fixed with 0.5% paraformaldehyde solution for 20 min. After washing, the stained cells were analyzed via two-color flow cytometry on a FACS Caliber using CellQuest software (BD Biosciences, San Diego, CA, USA).

### 4.7. Quantitative Reverse Transcription-Polymerase Chain Reaction (qRT-PCR)

Total RNA was isolated from lung tissue using TRIzol^®^ and 2 µg total RNA were reverse-transcribed into cDNA using a First-Strand cDNA Synthesis kit according to the manufacturer’s protocol (Amersham Pharmacia, Piscataway, NJ, USA). qRT-PCR was performed using a SYBR Green PCR Master Mix (Applied Biosystems, Foster City, CA, USA) with an Applied Biosystems 7500 Real-Time PCR system according to the manufacturer’s instructions. The primer and probe sequences are listed in Table 2.

### 4.8. Western Blot Analysis

Lung proteins were extracted using NE-PER^®^ Nuclear and Cytosolic Extraction Reagents (Thermo Scientific, Seoul, Korea). The proteins were then quantified using a PROMEASURE assay kit (iNtRON Biotechnology, Seoul, Korea), separated via 10% sodium dodecyl sulfate-polyacrylamide gel electrophoresis (SDS-PAGE), and transferred onto polyvinylidene difluoride membranes. The membranes were blocked with a blocking buffer containing 5% non-fat dry milk and 0.1% Tween-20 in Tris-buffered saline. The membranes were then incubated with specific primary antibodies against total caspase-3, cleaved caspase-3, NLR family pyrin domain containing 3 (NLRP3), procaspase-1, caspase-1, and β-actin at 4 °C overnight, followed by 1 h of incubation with horseradish peroxidase-conjugated secondary antibody. The bound antibodies were visualized via enhanced chemiluminescence, and the images were analyzed using the software ImageJ. β-Actin was used as an internal control.

### 4.9. Immunofluorescence Staining

Frozen lung tissues were cut into 20-µm-thick sections using a cryostat microtome (CM 3050S; Leica Microsystems, Wetzlar, Germany). The lung sections were fixed with 4% paraformaldehyde and 4% sucrose in PBS for 40 min, permeabilized with 0.5% Nonidet P-40 in PBS, and blocked with 2.5% horse serum and 2.5% bovine serum albumin for 16 h. Double immunofluorescence staining was performed by incubating the sections with an antibody targeting F4/80, IL-1α, interleukin-1 receptor-associated kinase (IRAK-1), TNF-α, and CXCL-2 at 4 °C overnight. Subsequently, a fluorescein-conjugated secondary antibody was added for 2 h, and nuclear staining was performed using 4′,6-diamidino-2-phenylindole (DAPI). The sections were visualized using an Eclipse Ti-E inverted fluorescence microscope (Nikon Instruments Inc., Mississauga, ON, Canada). The mean fluorescence intensity was quantified using images obtained from three independent experiments with the software ImageJ.

### 4.10. Histopathological Analysis of Lung and Tracheal Tissues

The lung and tracheal tissues were fixed in formalin, embedded in paraffin, and cut into 5-μm-thick sections. Lung tissue sections were stained with hematoxylin and eosin (H&E) and Masson’s trichrome stain. Lung and tracheal tissue sections were stained with Alcian blue/Periodic acid-Schiff stain (AB/PAS). Tissue damage in the airway epithelium was quantified by a blinded researcher using a 5-point (0–4) grading system, as previously described [26,27].

### 4.11. Statistical Analysis

Data are presented as mean ± standard error of the mean (SEM) and were analyzed using a one-way analysis of variance followed by Dunnett’s multiple comparison test using Prism v7.0 (GraphPad Inc., San Diego, CA, USA). Significant differences are denoted as ^#^ *p* < 0.05, ^##^ *p* < 0.01, and ^###^ *p* < 0.005 compared to the BALB/c-Nr (NC) group, and * *p* < 0.05, ** *p* < 0.01, and *** *p* < 0.005 compared to the PM10D-CTL group.

## 5. Conclusions

In conclusion, administration of OFI extracts inhibited inflammatory responses in the airways by reducing the numbers of immune cells and regulating the expression of chemokines and inflammatory cytokines. OFI extract treatment also effectively alleviated lung tissue damage in PM10D-induced airway inflammation. Treatment with a 50% ethanol extract of OFI was found to be the most effective. The findings of this study therefore suggest that OFI treatment significantly ameliorates PM10D-induced airway inflammation and tissue damage. OFI may be useful in preventing and treating respiratory diseases.

## Figures and Tables

**Table 1:** Effects of water and ethanolic extracts of OFI on airway immune cell number and neutrophilic airway inflammation in a PM10-induced airway inflammation model.

Cell Phenotype (FACS Analysis) (×10^4^ mL)			PM10D				
		NC	CTL	Dexa −3 mg/kg	OFI-W 200 mg/kg	OFI-30E 200 mg/kg	OFI-50E 200 mg/kg
Lymphocyte	Lung	33.08 ± 1.81	79.23 ± 14.64 ^##^	43.94 ± 2.18 *	65.61 ± 10.80	56.03 ± 18.89	65.82 ± 4.67
Neutrophils		14.63 ± 4.04	43.50 ± 7.81 ^##^	54.79 ± 6.58	35.68 ± 8.54	52.20 ± 1.97 *	11.09 ± 0.74 ***
CD3^+^/CD4^+^		25.10 ± 1.11	63.80 ± 14.24 ^##^	37.8 ± 2.52	51.37 ± 11.08	58.04 ± 13.81	31.07 ± 3.05
CD3^+^/CD8^+^		9.55 ± 1.09	24.23 ± 4.27 ^###^	21.36 ± 1.98	21.03 ± 3.74	25.41 ± 5.23	15.07 ± 0.44 *
Gr-1^+^/CD11b^+^		3.83 ± 0.35	38.35 ± 7.57 ^###^	13.86 ± 1.18 **	10.11 ± 1.38 **	11.96 ± 0.31 **	5.80 ± 0.32 ***
CD3^+^/CD4^+^	BALF	0.02 ± 0.02	56.00 ± 1.90 ^###^	3.77 ± 2.57 ***	27.00 ± 1.61 ***	28.45 ± 1.37 ***	16.96 ± 2.33 ***
CD3^+^/CD8^+^		0.01 ± 0.01	24.25 ± 2.90 ^###^	1.00 ± 0.28 ***	15.52 ± 3.24 *	27.33 ± 9.65	9.77 ± 2.09 ***
Gr-1^+^/CD11b^+^		0.08 ± 0.00	166.72 ± 1.80 ^###^	16.74 ± 0.98 ***	84.72 ± 23.55 **	95.46 ± 10.46 ***	33.5 ± 18.25 ***

NC: BALB/c normal control group; CTL: PM10D-induced control group; Dexa: 3 mg/kg dexamethasone-treated PM10D-induced group; OFI-W 200: PM10D-induced group treated with 200 mg/kg OFI-water extract, respectively; OFI-30E 200: PM10D-induced group treated with 200 mg/kg OFI-30% ethanol extract, respectively; OFI-50E 200: PM10D-induced group treated with 200 mg/kg OFI-50% ethanol extract, respectively. Data are expressed as means ± SEM, *n* = 8. ^##^ *p* < 0.01, ^###^ *p* < 0.005 vs. NC; * *p* < 0.05, ** *p* < 0.01, and *** *p* < 0.005 vs. CTL.

**Table 2:** The primer and probe sequences.

Gene	Sequence (5′-3′)
IL-6	Forward: TCCAGTTGCCTTCTTGGGAC Reverse: GTGTAATTAAGCCTCCGACTTG
IL-5	Forward: AGCACAGTGGTGAAAGAGACCTT Reverse: TCCAATGCATAGCTGGTGATTT
IL-1β	Forward: CAGGGTGGGTGTGCCGTCTTTC Reverse: TGCTTCCAAACCTTTGACCTGGGC
TNF-α	Forward: GGCTTTCCGAATTCACTGGAGCCT Reverse: CCCCGGCCTTCCAAATAAATACATTCATA
MUC5AC	Forward: AGAATATCTTTCAGGACCCCTGCT Reverse: ACACCAGTGCTGAGCATACTTTT
CXCL-1	Forward: CCGAAGTCATAGCCACAC Reverse: GTGCCATCAGAGCAGTCT
TRPV1	Forward: CATCTTCACCACGGCTGCTTAC Reverse: CAGACAGGATCTCTCCAGTGAC
MIP-2 (CXCL-2)	Forward: ATGCCTGAAGACCCTGCCAAG Reverse: GGTCAGTTAGCCTTGCCTTTG
COX-2	Forward: GGGTGTCCCTTCACTTCTTTCA Reverse: TGGGAGGCACTTGCATTGA
GAPDH-Probe	Applied Biosystems^®^ Mouse GAPDH Endogenous Control (VIC^®^/MGB Probe, 4352339E)

## Data Availability

Data is contained within the article or supplementary material.

## Supplementary Materials

The following are available online at https://www.mdpi.com/article/10.3390/plants11040520/s1, Figure S1: Scheme of PM10D sensitization and challenge protocol. Table S1: Effects of water and ethanolic extracts of OFI on airway immune cell number and neutrophilic airway inflammation in a PM10-induced airway inflammation model.
