# Supplementary material for: Opuntia ficus-indica Alleviates Particulate Matter 10 Plus Diesel Exhaust Particles (PM10D)—Induced Airway Inflammation by Suppressing the Expression of Inflammatory Cytokines and Chemokines
Source: Plants (Basel). 2022 Feb 14;11(4):520. doi: 10.3390/plants11040520 (PMC8877671; doi:10.3390/plants11040520)
Supplement: Supplementary file 1 [file plants-11-00520-s001.zip › plants-1533540-supplementary.pptx]

## Slide 1
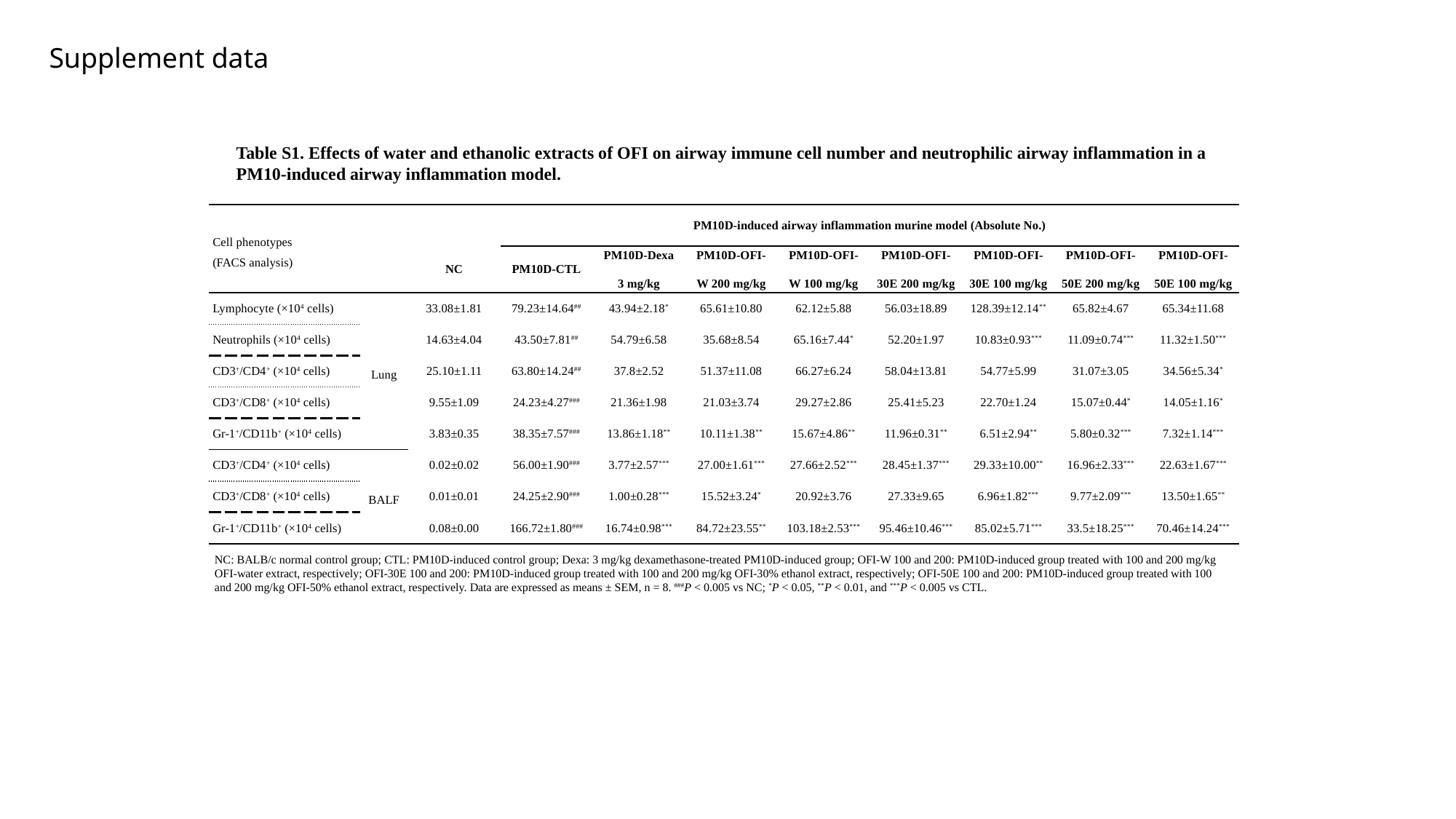

Supplement data
Table S1. Effects of water and ethanolic extracts of OFI on airway immune cell number and neutrophilic airway inflammation in a PM10-induced airway inflammation model.
| Cell phenotypes (FACS analysis) | | | PM10D-induced airway inflammation murine model (Absolute No.) | | | | | | | |
| --- | --- | --- | --- | --- | --- | --- | --- | --- | --- | --- |
| | | NC | PM10D-CTL | PM10D-Dexa 3 mg/kg | PM10D-OFI- W 200 mg/kg | PM10D-OFI- W 100 mg/kg | PM10D-OFI- 30E 200 mg/kg | PM10D-OFI- 30E 100 mg/kg | PM10D-OFI- 50E 200 mg/kg | PM10D-OFI- 50E 100 mg/kg |
| Lymphocyte (×104 cells) | Lung | 33.08±1.81 | 79.23±14.64## | 43.94±2.18\* | 65.61±10.80 | 62.12±5.88 | 56.03±18.89 | 128.39±12.14\*\* | 65.82±4.67 | 65.34±11.68 |
| Neutrophils (×104 cells) | | 14.63±4.04 | 43.50±7.81## | 54.79±6.58 | 35.68±8.54 | 65.16±7.44\* | 52.20±1.97 | 10.83±0.93\*\*\* | 11.09±0.74\*\*\* | 11.32±1.50\*\*\* |
| CD3+/CD4+ (×104 cells) | | 25.10±1.11 | 63.80±14.24## | 37.8±2.52 | 51.37±11.08 | 66.27±6.24 | 58.04±13.81 | 54.77±5.99 | 31.07±3.05 | 34.56±5.34\* |
| CD3+/CD8+ (×104 cells) | | 9.55±1.09 | 24.23±4.27### | 21.36±1.98 | 21.03±3.74 | 29.27±2.86 | 25.41±5.23 | 22.70±1.24 | 15.07±0.44\* | 14.05±1.16\* |
| Gr-1+/CD11b+ (×104 cells) | | 3.83±0.35 | 38.35±7.57### | 13.86±1.18\*\* | 10.11±1.38\*\* | 15.67±4.86\*\* | 11.96±0.31\*\* | 6.51±2.94\*\* | 5.80±0.32\*\*\* | 7.32±1.14\*\*\* |
| CD3+/CD4+ (×104 cells) | BALF | 0.02±0.02 | 56.00±1.90### | 3.77±2.57\*\*\* | 27.00±1.61\*\*\* | 27.66±2.52\*\*\* | 28.45±1.37\*\*\* | 29.33±10.00\*\* | 16.96±2.33\*\*\* | 22.63±1.67\*\*\* |
| CD3+/CD8+ (×104 cells) | | 0.01±0.01 | 24.25±2.90### | 1.00±0.28\*\*\* | 15.52±3.24\* | 20.92±3.76 | 27.33±9.65 | 6.96±1.82\*\*\* | 9.77±2.09\*\*\* | 13.50±1.65\*\* |
| Gr-1+/CD11b+ (×104 cells) | | 0.08±0.00 | 166.72±1.80### | 16.74±0.98\*\*\* | 84.72±23.55\*\* | 103.18±2.53\*\*\* | 95.46±10.46\*\*\* | 85.02±5.71\*\*\* | 33.5±18.25\*\*\* | 70.46±14.24\*\*\* |
NC: BALB/c normal control group; CTL: PM10D-induced control group; Dexa: 3 mg/kg dexamethasone-treated PM10D-induced group; OFI-W 100 and 200: PM10D-induced group treated with 100 and 200 mg/kg OFI-water extract, respectively; OFI-30E 100 and 200: PM10D-induced group treated with 100 and 200 mg/kg OFI-30% ethanol extract, respectively; OFI-50E 100 and 200: PM10D-induced group treated with 100 and 200 mg/kg OFI-50% ethanol extract, respectively. Data are expressed as means ± SEM, n = 8. ###P < 0.005 vs NC; *P < 0.05, **P < 0.01, and ***P < 0.005 vs CTL.

## Slide 2
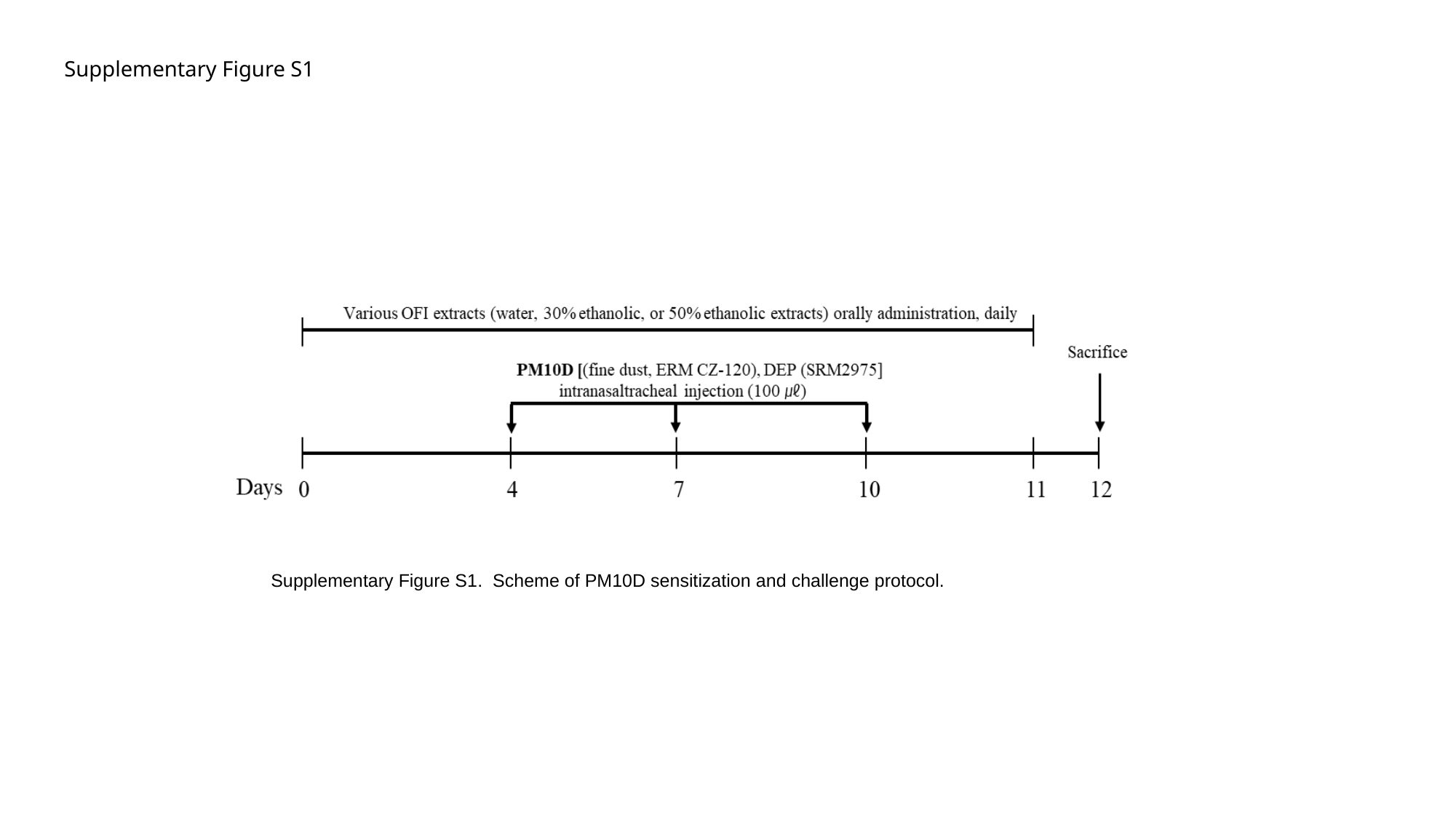

Supplementary Figure S1
Supplementary Figure S1. Scheme of PM10D sensitization and challenge protocol.
